# Proceedings of the Eleventh Annual UT-ORNL-KBRIN Bioinformatics Summit 2012

**DOI:** 10.1186/1471-2105-13-S12-A1

**Published:** 2012-07-31

**Authors:** Eric C Rouchka, Robert M Flight, Hunter NB Moseley

**Affiliations:** 1 Department of Computer Engineering and Computer Science, University of Louisville, Duthie Center for Engineering, Louisville, KY 40292, USA; 2 Department of Anatomical Sciences and Neurobiology, University of Louisville, Louisville, KY 40292, USA; 3 Department of Chemistry, Center for Regulatory and Environmental Analytical Metabolomics, University of Louisville, Louisville, KY 40292, USA

## 

The University of Tennessee (UT), the Oak Ridge National Laboratory (ORNL), and the Kentucky Biomedical Research Infrastructure Network (KBRIN), have collaborated over the past eleven years to share research and educational expertise in bioinformatics. One result of this collaboration is the joint sponsorship of an annual regional summit to bring together researchers, educators and students who are interested in bioinformatics from a variety of research and educational institutions. This summit provides unique opportunities for collaboration and forging links between members of the various institutions. This year, the Eleventh Annual UT-ORNL-KBRIN Bioinformatics Summit was held at the Seelbach Hilton Hotel in Louisville, Kentucky from March 30-April 1, 2012. A total of 232 participants pre-registered for the summit, with 126 from various Kentucky institutions and 80 from various Tennessee institutions. A number of additional participants came from universities and research institutions from other states and countries, e.g. University of Arkansas Medical Sciences, Michigan State University, University of Cincinnati, Iowa State University, etc. Eighty-four registrants were faculty, with an additional 68 students, 37 staff, and 32 postdoctoral participants (with 12 undeclared).

The conference program consisted of three days of presentations. The first day included a pre-summit of talks by Kentucky researchers and a workshop on Next-Generation Sequencing technologies. The next two days were dedicated to scientific presentations divided into four plenary sessions on Next-Generation Sequencing, Medical Informatics, Metagenomics, and Behavioral and Comparative Genomics. The Medical Informatics session was followed by four short talks, selected from 47 submitted poster abstracts.

## Pre-summit Kentucky session

Claire Rinehart (Western Kentucky University) and Jerzy Jaromczyk (University of Kentucky) organized a three-hour pre-summit session focused on bioinformatics research and education currently on-going in the Commonwealth of Kentucky. Eric Rouchka (University of Louisville) and Nigel Cooper (University of Louisville) welcomed everyone to the pre-summit Kentucky session. A series of short talks from various Kentucky researchers followed. The first talk, by Neil Moore (University of Kentucky) titled “Finding Long Protein Products of Alternative Spliced Genes” discussed a methodology on detecting alternatively spliced genes within next-generation sequence data by looking at long open reading frames in conjunction with canonical and alternative intron-exon boundaries [1]. Eric Rouchka followed with a talk “Systems Level Approach to Understanding Intercellular Interactions” that discussed two recently developed approaches, AbsIDConvert [http://bioinformatics.louisville.edu/abid/] and a Bioconductor package categoryCompare [http://www.bioconductor.org/packages/release/bioc/html/categoryCompare.html]. In addition, updates on educational resources within the Commonwealth of Kentucky were presented including “Training Platforms for Next Generation Sequencing Data” (Pat Calie, Eastern Kentucky University) which focused on a newly developed NSF EPSCoR summer program for training in Next-Generation Sequencing Technologies to be held at the University of Kentucky; “HHMI Science Education Alliance Experiment to Engage Freshmen in Genomic Research” (Claire Rinehart) which described the results of Western Kentucky’s participation in a Howard Hughes Medical Institute program that aims to involve undergraduates in scientific discovery through novel sequencing of mycobacteriophages [2]; “Interdisciplinary Courses and Courses for Interdisciplinary Students at UK” (Jerzy Jaromczyk), and “Development of a Ph.D. in Interdisciplinary Studies: Concentration in Bioinformatics” (Eric Rouchka) which highlighted the newly established bioinformatics Ph.D. program at the University of Louisville [http://bioinformatics.louisville.edu/phd/], the first program of its kind in the Commonwealth of Kentucky.

## Friday workshops

Matt Osentoski and Matt Dyer of Life Technologies (Carlsbad, CA; http://www.lifetechnologies.com) opened the Bioinformatics Summit with workshops discussing the next generation sequencing platforms and bioinformatics aspects offered by Life Technologies. A specific focus was placed on the Ion Torrent^TM^ sequencers that use a semiconductor platform to detect the current nucleotide incorporation by measuring the change in pH that results [3]**.** These include the Ion Personal Genome Machine^TM^ (PGM^TM^) which can use one of three chips, depending upon the desired coverage: The Ion 314^TM^ Chip (1 million wells, 10Mb output), Ion 316^TM^ Chip (6 million wells, 100Mb output), and 318^TM^ Chip (11 million wells, 1Gb output). The PGM^TM^ is best suited to small genomes and targeted gene sets based on the overall coverage. Newer technology to be released in 2012 includes the Ion Proton^TM^, which aims to get closer to the possibility of the $1,000 genome. The unreleased Proton I^TM^ Chip will contain 165 million wells and the Proton II^TM^ Chip will contain 660 million wells, which will allow for a genome the size of the human genome to be sequenced on a single chip. Matt Osentoski discussed the types of applications suitable to each platform as well as a detailed description of the first three steps of the Ion PGM^TM^ Sequencer Workflow: 1) library construction; 2) template preparation; and 3) sequencing. As a demonstration of the fast turnaround time, he discussed the use of the Ion PGM^TM^ in whole genome sequencing and characterization of an outbreak of *E. coli* O104:H4 strain in Germany associated with haemolytic uremic syndrome [4]. Matt Dyer focused on the fourth step (data analysis) of the workflow. This discussion was particularly aimed towards use of the Ion Torrent^TM^ Community, a social networking website consisting of over 7,000 registered users created for the purpose of aiding each other in technical sequencing issues as well as serving as a dissemination point for bioinformatics software developed specifically for the analysis of sequencing data generated by the Ion Torrent^TM^ platforms.

Jon Armstrong of Cofactor Genomics (St. Louis, MO; http://www.cofactorgenomics.com) followed with a discussion titled “Strategies for *De Novo* Assembly of Genomes and Transcriptomes Using Combined Illumina and Roche 454 Sequencing Data.” This presentation discussed the use of multiple sequencing approaches for genome and transcriptome construction by combining the benefit of sequencing depth provided by Illumina with the sequencing length obtained from Roche 454 sequencing. In addition to the benefits in assembly provided by this data, Jon discussed many of the pitfalls faced using a variety of assemblers, arising out of the difference in read coverage for these approaches.

## Session I: Next generation sequencing

Chris Ponting (University of Oxford) began the formal program with a talk titled “How Much DNA/RNA is Lineage-Specific, Noncoding and Functional?” In this presentation, Dr. Ponting discussed the fact that the majority of a genome is transcribed at one point or another [5], including long intergenic non-coding RNA (lincRNA) [6]. His group has shown that a large portion of the human genome (10%-15%) is likely to be functional [7] due to functional non-coding RNAs. These non-coding RNAs are shown to evolve at faster rates than coding RNAs [8] with lineage-specific gain or loss of function, as illustrated in the case of the zebra finch songbird [9]. Dr. Ponting discussed the extent to which lincRNA loci are retained or lost across multiple evolutionary lineages using a neutral insertion/deletion model and RNASeq data to identify functional sequence that is not conserved [6]. The results of his work in lineage specific models indicate that DNA and RNA sequence gains and/or loses function in a transient manner, with a half-life of 20 million years [10].

The second talk of the session, “Comparative RNA Sequencing Across Archaea Reveals a Constellation of New Small RNAs”, was presented on Saturday morning by Todd Lowe (University of California at Santa Cruz). This presentation focused on the use of comparative genomics and high-throughput RNA sequencing within the hyperthermophilic genus *Pyrobaculum* for detecting functional non-coding RNAs [11]. One of the main results of this work show that ncRNA gene families have a greater variation in sequence features than previously observed, causing computational methodologies to fail in detecting additional family members [12,13]. A second conclusion is that there are a large number of small RNA transcripts overlapping the 5’ and 3’ ends of genes which may play significant roles in regulation. A specific example with the transcription initiation factor B (TFB) in *Pyrobaculum *[14] was presented in detail, along with a discussion of the Archaeal Genome Browser [15,16].

## Session II: Medical informatics

Paul Harris (Vanderbilt University) led off the Medical Informatics session with a presentation on the Research Electronic Data Capture (REDCap) system developed at Vanderbilt University for providing an infrastructure support for translational research [17]. REDCap has over 350 active institutional partners around the globe, and has been employed in over 33,000 projects. The platform has evolved into a community-based system that provides end users with a secure web application to support data capture for research studies. Dr. Harris provided a brief introduction into the use of REDCap. He illustrated the power REDCap has to facilitate both basic and clinical research, particularly in locations where clinical and translational research is beginning to emerge, such as Clinical and Translational Science Awards (CTSA).

Dr. Todd Johnson (University of Kentucky) followed with a presentation “Biomedical Informatics for Clinical and Translational Science at the University of Kentucky.” In this presentation, Dr. Johnson gave an excellent overview of the history of biomedical informatics [18], explaining that the deluge of high dimensional “big data” has made the use of bioinformatics and biomedical informatics a necessity for data interpretation [19]. Dr. Johnson also discussed many of the projects on-going in the newly formed Division of Biomedical Informatics at the University of Kentucky, including a project to automatically code key cancer concepts from electronic pathology reports, and a project to help predict and reduce the number of readmissions to acute care hospitals.

## Session III: Metagenomics

Janet Jansson (Lawrence Berkeley National Laboratory) led the Metagenomics session with a plenary talk titled “Illumination of Soil Microbial Community Functions using Metagenomics.” In this presentation, Dr. Jansson focused on two current soil metagenomics projects. In the first portion of her talk, Dr. Jansson focused on the results of their study on an Alaskan permafrost microbial community [20]. The results of next-generation sequencing analysis on intact core samples using 16S sequencing for identification of microbes and construction of a 1.9 Gb methanogenome using Illumina sequencing revealed a rapid shift in microbial and functional gene abundances in the transition from frozen to thawed states. Many of these genes appear to be involved in carbon and nitrogen cycling, suggesting the role that rising temperatures have on the release and processing of methane trapped in permafrost and subsequently consumed by methanotrophic bacteria. After a one week period, the metagenomes appear to stabilize comparatively to one another. The second portion of her talk focused on a pilot project for JGI’s Soil Metagenome Initiative [21] for the Great Prairie Metagenome. This project is the largest environmental metagenomics project to date, producing nearly two terabases of data for studying the impact of cultivation on metagenomes within the Great Prairie.

## Session IV: Behavioral and comparative genomics

Hans Hofmann began the Sunday session with a plenary talk titled “Gene Modules, Neural Circuits and Social Networks: Integrating Complex Data Across Levels of Organization and Over Evolutionary Time.” In this entertaining presentation, Dr. Hofmann presented an integrated approach to understanding the evolution of social behavior in terms of challenges and opportunities an organism faces using combinations of observed behavior, hormone profiles, gonadal histology, and gene expression [22,23]. He demonstrated the necessity for this integrated approach while presenting results in terms of observations of social competition in both male and female African cichlid fish [24-29].

Elissa Chesler (The Jackson Laboratory) closed out the 2012 Summit with the plenary talk “Accelerating Discovery in Behavioral Genetics Through Integrative Genetics and Genomics” [30]. This presentation focused on the integrative use of phenotype-specific information to determine Quantitative Trait Loci (QTLs) and candidate genes that may be of interest for further interrogation. Dr. Chesler focused on the use of GeneWeaver [31] and the Ontological Discovery Environment [http://ontologicaldiscovery.org] [32] for complex trait analysis. GeneWeaver provides integrative methodologies for enabling scientific discovery across disparate datasets such as genome-wide association studies, QTLs, microarrays, RNA-sequencing, and mutant phenotyping while the Ontological Discovery Environment provides statistical methodologies and visualizations for phenotypic-centered gene data. These tools used in conjunction with one another provide an avenue for exploring existing datasets using a phenotype-based model.

## Posters and short talks

The poster session was held on day two. Forty-seven posters were on display, from a variety of different research areas. A number of posters were also selected for short talks. These included “Delivering informatics for clinical research in developing countries” (Jonathan Babbage, Michigan State University); “QTLs for bone mineral density of femurs and tibias from recombinant inbred strains derived from C57BL/6J and DBA/2J inbred strains” (Lishi Wang, UTHSC); “A linear framework for transcript quantification from RNA-seq data” (Jinze Liu, University of Kentucky); and “AbsIDconvert: An absolute approach for converting genetic identifiers at different granularities” (Fahim Mohammad, University of Louisville).

For full author lists and abstracts see the rest of the supplement.

## Future plans

The 2013 Bioinformatics summit will return to the state of Tennessee in the spring of 2013. Potential focus areas include current technological trends in molecular biology, applications of next-generation sequencing, and systems biology.
